# Colocalization of Tectal Inputs With Amygdala-Projecting Neurons in the Macaque Pulvinar

**DOI:** 10.3389/fncir.2018.00091

**Published:** 2018-10-24

**Authors:** Catherine Elorette, Patrick A. Forcelli, Richard C. Saunders, Ludise Malkova

**Affiliations:** ^1^Interdisciplinary Program in Neuroscience, Georgetown University School of Medicine, Washington, DC, United States; ^2^Department of Pharmacology and Physiology, Georgetown University School of Medicine, Washington, DC, United States; ^3^Department of Neuroscience, Georgetown University School of Medicine, Washington, DC, United States; ^4^Laboratory of Neuropsychology, National Institute of Mental Health (NIMH), Bethesda, ML, United States

**Keywords:** subcortical, superior colliculus, blindsight, retrograde, anterograde, anatomy

## Abstract

Neuropsychological and neuroimaging studies have suggested the presence of a fast, subcortical route for the processing of emotionally-salient visual information in the primate brain. This putative pathway consists of the superior colliculus (SC), pulvinar and amygdala. While the presence of such a pathway has been confirmed in sub-primate species, it has yet to be documented in the primate brain using conventional anatomical methods. We injected retrograde tracers into the amygdala and anterograde tracers into the colliculus, and examined regions of colocalization of these signals within the pulvinar of the macaque. Anterograde tracers injected into the SC labeled axonal projections within the pulvinar, primarily within the oral, lateral and medial subdivisions. These axonal projections from the colliculus colocalized with cell bodies within the pulvinar that were labeled by retrograde tracer injected into the lateral amygdala. This zone of overlap was most notable in the medial portions of the medial (PM), oral (PO) and inferior pulvinar (PI), and was often densely concentrated in the vicinity of the brachium of the SC. These data provide an anatomical basis for the previously suggested pathway mediating fast processing of emotionally salient information.

## Introduction

Threatening stimuli require fast detection and response from an organism. The canonical cortical visual processing stream refines information from coarse, low level representations in early cortical areas to detailed, high level information. The “trade off” for this refinement is that it requires many synaptic connections. Conserved patterns of low-level visual information, e.g., something moving quickly in peripheral vision, may be sufficient to serve as a threat detection pathway (Dean et al., 1989; Soares et al., 2017). On the basis of behavioral and anatomical studies some have suggested that low level or coarse visual information may travel to the forebrain in a rapid, subcortical manner (LeDoux, 1996; Silverstein and Ingvar, 2015).

Despite a lack of conscious recognition, patients with damage to primary visual cortex can retain appropriate responses to visual stimuli (Pöppel et al., 1973; Richards, 1973; Sanders et al., 1974; Weiskrantz et al., 1974). In a non-conscious manner, patients with so-called “blindsight” can detect, localize, and distinguish between stimuli that are presented within the field of vision damaged by a lesion (Cowey and Stoerig, 1991; Sahraie et al., 1998). Similarly, even after total destruction of V1, non-human primates can track moving stimuli and distinguish between different textures, colors, shape, or frequency of presentation of stimuli (Weiskrantz, 1963; Humphrey and Weiskrantz, 1967; Mohler and Wurtz, 1977; Miller et al., 1980). The preservation of visual processing in blindsight is subserved in part by projections from the retina to the lateral geniculate nucleus (LGN) of the thalamus (Warner et al., 2010). The LGN, which is the primary relay to visual cortex, projects not only to primary visual areas, but also to higher order cortical regions (e.g., V4, MT; Born and Bradley, 2005). These extrastriate projections are likely preserved after damage to V1 (Schmid et al., 2009). In addition, the superior colliculus (SC), which receives direct retinal input, may provide a relay for visual processing in the absence of V1. Accordingly, inactivation of either the LGN or the SC disrupts blindsight in macaques (Schmid et al., 2010; Kato et al., 2011; Takakuwa et al., 2017).

In addition to the preservation of these non-conscious visual abilities, processing of emotional information in patients with blindsight is likewise preserved. For example, patients can continue to discriminate between facial expressions (de Gelder et al., 1999) or fear-evoking images (Morris et al., 2001) after damage to V1. Furthermore, activation of the amygdala in response to emotionally salient visual stimuli has been observed using fMRI in cortically blind individuals (Vuilleumier et al., 2002). Consistent with the notion that rapid detection of threatening stimuli may rely on a fast (subcortical) relay, in intact individuals masked presentation of emotionally salient stimuli likewise activates the amygdala in the absence of conscious awareness (Whalen et al., 1998; Liddell et al., 2005). While the LGN likely mediates many features of blindsight as described above, other subcortical structures have been suggested to mediate the fast processing of emotionally salient information. Notably, both the SC and pulvinar display coincident activation with the amygdala during processing of emotional stimuli (Morris et al., 1999; Koller et al., 2018); together with the amygdala, these structures have been suggested to serve as a coarse, fast, subcortical pathway for the detection and response to emotionally salient visual information (Weiskrantz et al., 1974; LeDoux, 1996).

The SC in primates receives input from ~10% of retinal ganglion cells, and detects stimuli both in the central and peripheral field of vision (Perry and Cowey, 1984; Hofbauer and Dräger, 1985); in this way the SC may be optimally suited for detection of approaching threats. While the SC does not project directly to the amygdala, there is a well-documented projection from the SC to the pulvinar (Gattass et al., 2018, for review). Both the superficial and deep layers of SC project to pulvinar, including to the inferior (Stepniewska et al., 2000) medial, lateral and oral regions (Benevento and Fallon, 1975; Trojanowski and Jacobson, 1975; Benevento and Standage, 1983). Likewise, a projection from the pulvinar to the amygdala has been described (Locke, 1960; Jones and Burton, 1976; Aggleton et al., 1980; Norita and Kawamura, 1980; Romanski et al., 1997; Stefanacci and Amaral, 2000) with the majority of this projection targeting the lateral nucleus. This finding is consistent with previous findings that the bulk of subcortical and cortical inputs in the amygdala enter through the lateral nucleus (Amaral et al., 1992), considered the main input nucleus of the amygdala. Neuroimaging studies in both human and macaque subjects have shown probabilistic connectivity among SC, pulvinar and amygdala (Tamietto et al., 2012; Rafal et al., 2015) using diffusion tensor imaging (DTI) and tractography methods. However, it remains to be determined if the regions of the pulvinar that project to the amygdala are the same regions that receive input from the SC. In the present study, we aimed to address this gap. To achieve this goal, we injected anterograde tracers in the SC and retrograde tracers in the lateral nucleus of the amygdala and documented an overlap between retrograde and anterograde labeling within the pulvinar.

## Materials and Methods

### Subjects

Two male macaque monkeys (1 *Macaca mulatta* (Y), 1 *Macaca nemestrina* (S)) were used for these experiments. Animal Y was 4 years old and weighed 8.2 kg and Animal S was 6 years old and weighed 17.4 kg at the time of surgery. MRI-guided injections of anatomical tracers were performed stereotaxically under sterile conditions. Injections sites, tracers used and volumes of injection for each case are shown in Table 1. The animals received bilateral injections of anterograde tracer (fluoroemerald, FE) in SC and retrograde tracer (choleratoxin B, CTB) in the lateral nucleus of the amygdala; this yielded four cases for anatomical analysis. This study was carried out in accordance with the recommendations of the Guide for the Care and Use of Laboratory Animals. The protocol was approved by the Georgetown University Animal Care and Use Committee.

**Table 1:** Tracer injections across cases.

Case	Tracer	Supplier	Site	Volume (μl)
Y1	Choleratoxin B	List Labs	L Amygdala	2
	Fluoroemerald	Invitrogen	L SC	2.3
Y2	Choleratoxin B	List Labs	R Amygdala	2.5
	Fluoroemerald	Invitrogen	R SC	2.3
S1	Choleratoxin B AlexaFluor-594 conjugate	Invitrogen	L Amygdala	2.6
	Fluoroemerald	Invitrogen	L SC	2
S2	Choleratoxin B	List Labs	R Amygdala	1.8
	Fluoroemerald	Invitrogen	R SC	2

### MRI, Surgery and Injections

To obtain a pre-operative MRI scan, each animal was sedated with ketamine (10 mg/kg), intubated, and maintained at a stable plane of anesthesia using isoflurane (1%–4%). Animals were then transported to the imaging facility (Center for Molecular Imaging at Georgetown University Medical Center) where they were placed into a standard MRI-compatible stereotaxic frame. Each animal received a T1-weighted MRI scan using a custom surface coil in Siemens Trio 3T MRI scanner, as previously described (Wellman et al., 2005). An MPRAGE pulse sequence (TR = 1,600 ms, TE = 4.38 ms, TI = 640 ms, flip angle = 15 degrees, averages = 3, FOV = 256 × 256 mm^2^) was used to acquire a 3D volume of the monkey brain with an effective resolution of 1.0 mm^3^. The resulting subject-specific atlas was used to calculate injection coordinates relative to the earbar and the superior sagittal sinus, both of which were visible on the scans (Saunders et al., 1990).

After the MRI scan, each animal was transported to the surgical suite while remaining in the stereotaxic frame. During surgery, vital signs (heart rate, respiratory rate, body temperature, oxygen saturation, electrocardiogram and end-tidal CO_2_) were monitored. Body temperature was maintained using a heated table and blankets. Intravenous fluids (lactated Ringer’s solution) were delivered during the surgery.

The skin and galea were opened in anatomical layers to expose the cranium. A midline craniotomy (approximately 4 by 6 mm) was placed at the anteroposterior level of the intra-aural plane to expose the superior sagittal sinus, which served as the midline reference point. Craniotomies were placed above the amygdala and SC (three in total), the dura was incised, and a Hamilton syringe (30 gauge) was lowered to the MRI-derived coordinates. Injections were performed at a rate of 0.2 μL/min, and the syringe was left in place for a minimum of 10 min following injection to prevent reflux of the tracer up the needle tract. At the conclusion of the injections, the dura, galea and skin were closed in anatomical layers. Post-operatively, animals received antibiotics, analgesics and dexamethasone (0.5–1 mg/kg) in consultation with the facility veterinarians.

### Tissue Preparation

After a survival period of 14 days for Animal Y and 15 days for Animal S, the animals were deeply anesthetized with a sodium pentobarbital-based euthanasia solution and perfused transcardially with phosphate buffered saline followed by 4% paraformaldehyde. Brains were removed from the skull and post-fixed overnight in 4% paraformaldehyde. Following post-fixation, brains were cryoprotected in a solution of 10% glycerol and 2% DMSO in phosphate buffer for 24 h, then transferred to a 20% glycerol, 2% DMSO solution in phosphate buffer for 48 h (Rosene et al., 1986). Cryoprotected brains were blocked in the coronal plane and then flash frozen in −80°C isopentane and stored at −80°C until sectioning. The tissue was cut coronally in sections of 40 μm thickness on a freezing stage sliding microtome (American Optical Model 860 Microtome Physitemp Instruments Inc., BFS-40MP Freezing stage).

### Histological Procedures

Every 10th section from each brain was mounted onto gelatin subbed slides and air dried. The mounted sections were processed through a series of ethanol solutions, defatted, stained with thionin and cover slipped using DPX mountant (Sigma-Aldrich, St. Louis, MO, USA). These thionin stained sections were used to visualize the subdivisions of the thalamic nuclei, amygdala and SC.

To visualize CTB and FE labeling a series of sections was processed for each case through the pulvinar; adjacent sections were spaced by ~400 μm. The antibodies, suppliers and dilutions used for immunohistochemical and immunofluorescent staining procedures are listed in Table 2.

**Table 2:** Antibodies used for immunofluorescence and immunohistochemistry.

Antibody	Supplier Catalog #	Type	Host	Dilution	Immunogen
Anti-CTB	List Biological #703	Polyclonal	Goat	1:3,200	B subunit (choleragenoid)
Anti-Goat AlexaFluor-594 conjugated secondary	Jackson Immunoresearch #705-586-147	IgG (H + L)	Donkey	1:2,000	Goat IgG
Anti-goat HRP-conjugated secondary	Jackson Immunoresearch #705-005-147	IgG (H + L)	Donkey	1:200	Goat IgG
Anti-FE	ThermoFisher #A-11095	Polyclonal	Rabbit	1:4,000	Fluorescein
Anti-rabbit AlexaFluor-488 conjugated secondary	Jackson Immunoresearch #705-545-003	IgG (H + L)	Donkey	1:2,000	Rabbit IgG

### Immunofluorescence

The sections were washed in phosphate-buffered saline (PBS) containing 0.3% TX-100 three times for 5 min and placed in blocking solution with agitation for 1 h. Blocking solution consisted of 2% bovine serum albumin, 3.75% normal goat serum, 0.3% TX-100, all in PBS. Primary antibodies (anti-CTB and anti-FE, described in Table 2), were added to the blocking solution and the tissue was incubated for an additional 48 h at 4°C. The sections were washed five times for 5 min, then placed in secondary antibody solution (donkey anti-goat Alexa 594 and donkey anti-rabbit Alexa 488, described in Table 2), which consisted of 2% BSA, 0.3% TX-100, and 3.75% normal goat serum, all in PBS. The sections were incubated in secondary antibody for 90 min with agitation at room temperature. Following incubation in secondary antibody, sections were washed with PBS five times for 5 min each, mounted on gelatin-coated slides, briefly air dried, and coverslipped with Vectashield hard set with DAPI (Vector Laboratories). This process was used for cases Y1, Y2 and S2. In case S1, two 1-in-10 series through the pulvinar were stained separately. In one series, axons projecting from the SC to the pulvinar were visualized via immunofluorescence as described above, using only the antibodies (anti-FE) corresponding to the tracer injected in SC. The second series was processed for immunohistochemistry (described below).

For all cases, sections through the amygdala and SC were processed as described above to visualize the injection sites. The procedure was the same as that described for immunofluorescence above, but only the antibodies that corresponded to the injection sites were used (anti-CTB in the amygdala and anti-FE in the SC).

### Immunohistochemistry

For one case (S1), visualization of the CTB was enhanced using immunohistochemistry. Sections were washed in PBS containing 0.3% Triton X-100 (TX-100) three times for 5 min, quenched in 0.6% peroxide and PBS solution for 10 min, and placed in blocking solution with agitation for 1 h. Blocking solution consisted of 2% bovine serum albumin, 3.75% normal goat serum, 0.3% TX-100, all in PBS. Primary antibody (anti-CTB, described in Table 2) was added to the blocking solution and the tissue was incubated for an additional 48 h at 4°C. The sections were washed five times for 5 min, then placed in secondary antibody solution (Biotin-SP conjugated donkey anti-goat, described in Table 2) and incubated with agitation for 90 min at room temperature. These sections were washed five times for 5 min in PBS, placed in ABC solution (Vector Labs) for 90 min with agitation, washed five times for 5 min, and placed in diaminobenzidine tetrachloride (DAB, Sigma Aldrich, St. Louis, MO, USA) solution for 10 min with agitation. Then 0.025% peroxide was added and the sections were allowed to stain for approximately 3.5 min until there was visible contrast in the tissue. The sections were then washed with PBS five times, mounted on gelatin-coated slides, air-dried and dehydrated through ascending concentrations of ethanol before being cleared in xylenes and coverslipped with DPX.

### Microscopy and Data Analysis

**Figure 1:**
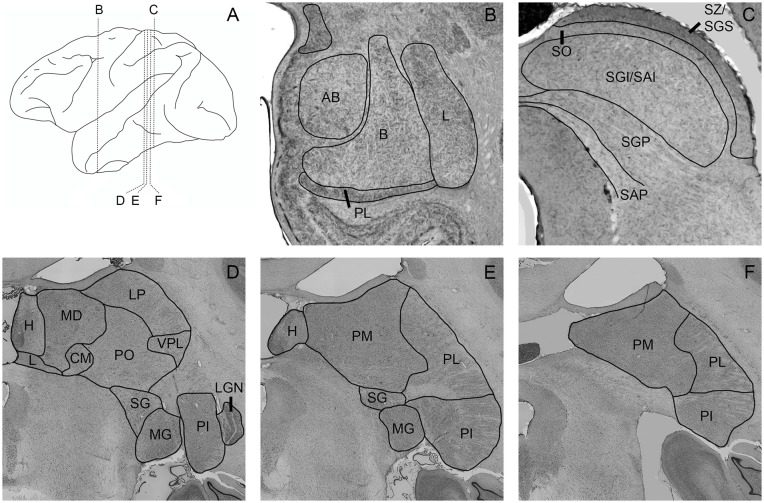
Photomicrographs of nissl-stained sections through the amygdala, superior colliculus (SC), and pulvinar. Panel **(A)** shows the location of panels **(B–F)** within the brain. Amygdala nuclei **(B)** are outlined and labeled according to Amaral et al. (1992). AB, accessory basal; B, basal; L, lateral; M, medial; PL, paralaminar. SC nuclei **(C)** are outlined and labeled according to May (2006). SZ/SGS, stratum zonale/stratum griseum superficiale; SO, stratum opticum; SGI/SAI, stratum griseum intermedium/stratum album intermedium; SGP, stratum griseum profundum; SAP, stratum album profundum. Thalamic nuclei **(D–F)** are outlined and labeled according to the delineations of the Olszewski (1952). CM, centromedial nucleus; H, habenula; L, nucleus limitans; LGN, lateral geniculate nucleus; LP, lateral posterior nucleus; MD, mediodorsal nucleus; MG, medial geniculate nucleus; PI, inferior pulvinar; PL, lateral pulvinar; PM, medial pulvinar; PO, oral pulvinar; SG, suprageniculate nucleus; VPL, ventroposteriolateral nucleus.

To outline the boundaries of individual subdivisions of the pulvinar, amygdala, and SC, we used thionin stained sections from a normal macaque brain (Figure 1). These sections were photographed using a Nikon NiE-E research microscope. Images were uniformly edited for brightness and noise reduction. The boundaries of amygdala nuclei and layers of the SC follow the divisions of Amaral et al. (1992) and May (2006), respectively. The boundaries of thalamic subnuclei were drawn following the conventions of the macaque thalamus atlas of Olszewski (1952). For further discussion of inferior pulvinar (PI) divisions, see Gutierrez et al. (1995).

**Figure 2:**
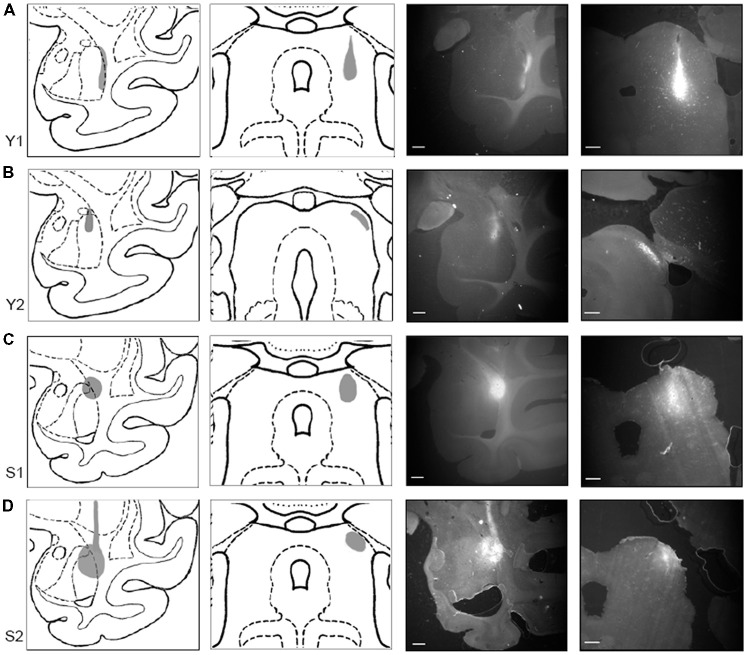
Tracer injections localized in SC and amygdala. Injection sites for cases Y1 **(A)**, Y2 **(B)**, S1 **(C)**, and S2 **(D)**. Each injection site is shown (in gray) on drawings of coronal sections through a standardized rhesus macaque atlas at the level of the amygdala (1st column) and the SC (2nd column). Images on the right show photomicrographs of the fluorescent tracer injections in amygdala (choleratoxin B (CTB); 3rd column; scale bar = 5 mm) and SC (fluoroemerald (FE); 4th column; scale bar = 1 mm).

To document the sites of tracer injections, immunofluorescent sections through the amygdala and the SC were photographed using a stereo microscope (Omano, China). Resulting micrographs were adjusted for brightness and contrast in Photoshop CC 2017 (Adobe) and are presented in Figure 2 together with drawings of matching sections from a standard rhesus macaque brain atlas generated in the Laboratory of Neuropsychology (LN), at the National Institute of Mental Health (NIMH).

**Figure 3:**
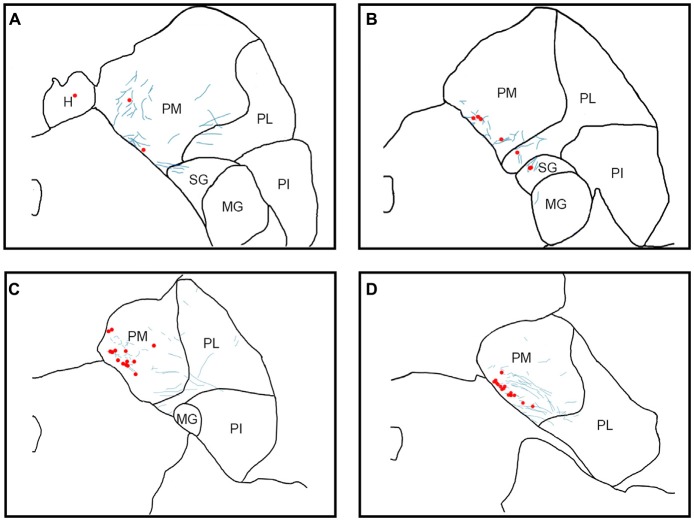
Pattern of overlapping label in Case Y1. Each panel **(A–D)** presents a plot of labeled cell bodies or fibers from different rostro-caudal levels through the pulvinar. Anterograde label (putative axons) after injection of FE in the SC are shown in cyan. Retrograde label (cell bodies) after injection of CTB in the amygdala are shown in red. Abbreviations for thalamic nuclei are as in Figure 1.

**Figure 4:**
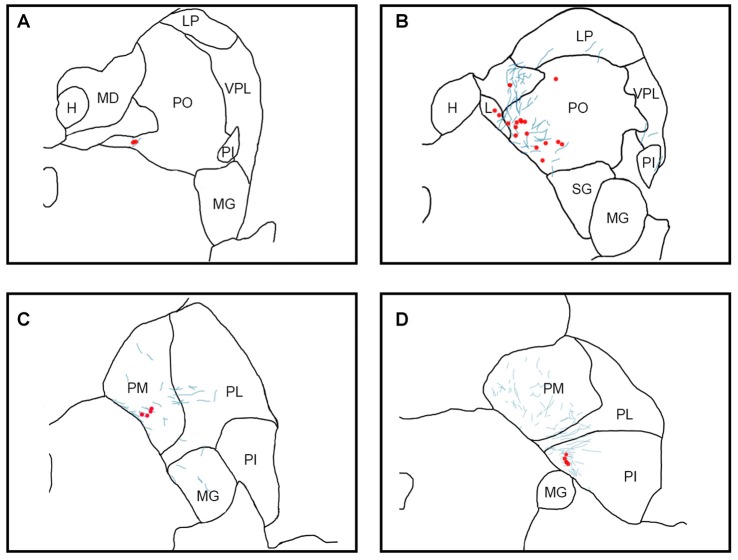
Pattern of overlapping label in Case Y2. Each panel **(A–D)** presents a plot of labeled cell bodies or fibers from different rostro-caudal levels through the pulvinar. Anterograde label (putative axons) after injection of FE in the SC are shown in cyan. Retrograde label (cell bodies) after injection of CTB in the amygdala are shown in red. Abbreviations for thalamic nuclei are as in Figure 1.

**Figure 5:**
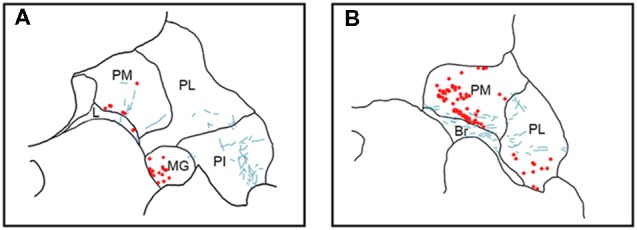
Pattern of overlapping label in Case S1. Each panel **(A,B)** presents a plot of labeled cell bodies or fibers from different rostro-caudal levels through the pulvinar. Anterograde label (putative axons) after injection of FE in the SC are shown in cyan. Retrograde label (cell bodies) after injection of CTB in the amygdala are shown in red. Abbreviations for thalamic nuclei are as in Figure 1. Br, brachium of the superior colliculus.

**Figure 6:**
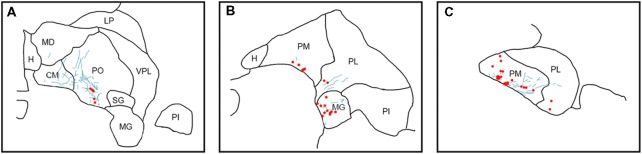
Pattern of overlapping label in Case S2. Each panel **(A–C)** presents a plot of labeled cell bodies or fibers from different rostro-caudal levels through the pulvinar. Anterograde label (putative axons) after injection of FE in the SC are shown in cyan. Retrograde label (cell bodies) after injection of CTB in the amygdala are shown in red. Abbreviations for thalamic nuclei are as in Figure 1.

Using sections processed for immunofluorescence (cases Y1, Y2, S2), labeled cell bodies and fibers (Figures 3, 4, 6) were localized using a Zeiss Axiophot microscope fitted with an MDPlot digitizer and software (Accustage, Shoreview, MN, USA). The outline of each section was traced and the individual axons and cell bodies labeled with the different tracers were plotted. Cell bodies labeled with CTB within the pulvinar were differentiated from non-specific background signal on the basis of distinctive color, luminance and morphology. Labeled cells were brighter than background signal and had a characteristic nuclear exclusion of fluorescence. The plotted sections were exported to Illustrator CS (Adobe) and aligned with images of the adjacent thionin stained section. In case S1, DAB staining of cell bodies in the pulvinar and fluorescent staining of axons from SC were marked as described above, then overlaid onto each other. These merged images were overlaid onto their corresponding thionin sections (Figure 5). In all figures, the plotted sections were cropped to show only the relevant regions. Labeling outside of the thalamus is not shown.

**Figure 7:**
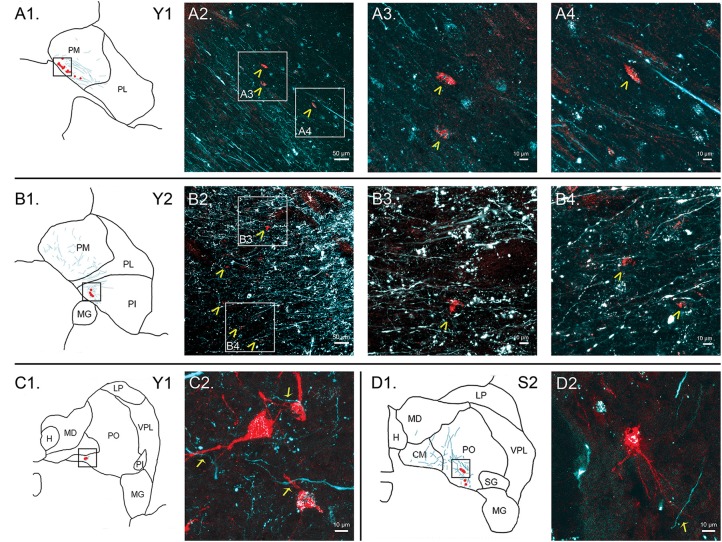
Colocalization of retrograde and anterograde labeling in the pulvinar. FE-labeled fibers resulting from injection into the SC are shown in cyan. CTB-immunoreactive cell bodies resulting from injection into the amygdala are shown in red. Atlas pictures are included to illustrate where each photo was taken. The case from which the images are derived is shown in the upper right corner. **(A)** Images of cells within PM, case Y1. Atlas image is shown in **(A1)**. Low power confocal image (20× magnification, **(A2)**) showing a band of labeled cell bodies (red) within the field of labeled axons running along the medial boundary of the PM, parallel to the brachium of the SC. Atlas image is shown in **(B1)**. Chevrons indicate cell bodies. Panels **(A3,A4)** are higher power (60×) magnifications of the areas indicated by boxes in **(A2)**. **(B)** Images of cells within PI, case Y2. Atlas image is shown in **(B1)**. Low power (20×, **(B2)**) confocal image showing colocalization of labeled cell bodies and axons in PI. Chevrons indicate cell bodies. Panels **(B3, B4)** show high power (60×) magnification. As in **(A)**, chevrons in B3 and B4 indicate areas of overlap in labeled cell bodies and axons. **(C)** Cells within PO, case Y1. Atlas image shown in **(C1)**. High power (63×) confocal image showing individual cell bodies and finer axonal processes in close proximity in **(C2)**. **(D)** Cells within PO, case S2. Atlas image shown in **(D1)**. High power (63×) confocal image in **(D2)**. Arrows indicate close associations between labeled terminal boutons and labeled cells. Scale bars are as indicated in each panel.

Sections through the pulvinar containing both retrogradely labeled cell bodies from tracers in the amygdala and axons labeled by anterograde tracers placed in the SC were acquired using a confocal microscope (Leica SP8) with 20× and 63× lenses (Leica HC PL APO 20 ×/0.75 IMM CORR CS2, Leica HC PL APO 63×/1.40 OIL CS2). Z-stacks were acquired for each region of interest, and maximal Z-projections were then pseudocolored and adjusted for brightness and contrast using ImageJ. Brightness and contrast were separately adjusted for each channel and each image, but all processes were applied to the *entire* image. Representative examples are presented in Figure 7.

## Results

### Injection Sites

In Case Y1 (Figure 2A), the CTB injection site in the amygdala extended along the lateral border of the lateral nucleus. Tracer extended into the white matter at the dorsal aspect of the injection. The dorsoventral extent of this injection spanned the majority of the lateral nucleus. The corresponding injection site in SC was localized to the lateral portion of stratum griseum intermedium (SGI), stratum album intermedium (SAI), stratum griseum profundum (SGP) and stratum album profundum (SAP). Together, these layers constitute the intermediate and deep layers of the SC. This injection site was the most ventral of the four cases.

In Case Y2 (Figure 2B), the CTB injection site in the amygdala was localized primarily to the dorsal portion of the lateral nucleus. The corresponding injection site in the SC was localized to the lateral stratum opticum (SO), with some tracer extended in to the stratum griseum superficiale (SGS) and SGI. The medial-lateral spread of the tracer was limited with most the tracer located immediately lateral to the brachium.

In Case S1 (Figure 2C), the CTB injection site in the amygdala was localized to the dorsolateral portion of the lateral basal nucleus and the dorsal portion of the lateral nucleus. The injection site extended beyond the boundary of the amygdala into the laterally adjacent white matter. The FE SC injection site was centered on the intermediate layers of SC with tracer extending into the superficial (SO, SGS) and stratum zonale (SZ) and deep layers. The SC injection site in this case was the most medial of the four cases.

In Case S2 (Figure 2D), the CTB injection site in amygdala was centered in the dorsal aspect of the lateral nucleus. The injection covered the dorsal half of the lateral nucleus and extended medially into the dorsal part of the lateral basal nucleus, with some coverage of the lateral adjacent white matter. The SC injection site was localized to the lateral SC and extended throughout the superficial and intermediate layers. The injection site, while lateral, did not extend into the brachium.

### Localization of Anterograde and Retrograde Label Within the Pulvinar

In Case Y1 (Figure 3), labeling of SC axons extended through the SC and spread through the medial pulvinar (PM), lateral pulvinar (PL) and suprageniculate (SG). Several fiber bundles followed along the brachium of the SC, with the lateral extent along the borders of the thalamic nuclei. Retrogradely labeled cell bodies from injections in the amygdala were present along the brachium, in the PM, the PL, and within the SG nucleus, and were concentrated to the ventromedial aspects of these regions. Areas of both anterogradely labeled axons (from SC) and retrogradely labeled cell bodies were observed medially, in medial and (sparsely) in PL (Figures 3B–D, 7A).

In Case Y2 (Figure 4), labeled axons from anterograde injections in SC extended through the SC into the oral pulvinar (PO) and mediodorsal thalamus, and further extended to the lateral posterior (LP) and ventroposteriolateral (VPL) nuclei of the thalamus. These fibers were localized to the ventromedial PM and the lateral portion of the mediodorsal thalamus. Retrogradely labeled cell bodies were present in PO, PM, PI and nucleus limitans. These labeled cells were located in close proximity to the brachium, although a few cells were observed in the dorsal PO. Areas of overlapping anterograde and retrograde labeling were observed in oral, medial and PI, concentrated ventromedially in these regions (see Figure 7B for an example of labeling within PI).

In Case S1 (Figure 5), labeled axons extended through the SC and laterally from the brachium into medial, lateral and PI. The tracts largely appeared to travel mediolaterally and were densest in the ventral aspect of the PM and the lateral aspect of the PI. Retrogradely labeled cell bodies resulting from injection in the amygdala were present in medial and PL, and to a lesser extent within the SG/medial geniculate (MG) nucleus. Most cell bodies were ventromedially localized, but there were a large number present in PM that extended dorsoventrally to cover approximately half of the area of the nucleus. Areas of overlap between the labeled axons and cell bodies were not directly observed due to the type of staining utilized in this case (see “Materials and Methods” section), but both labeled axons from the SC and labeled neurons projecting to the amygdala were present in the ventromedial aspect of the PM. The more extensive retrograde signal in this case may be due to the better signal-to-noise of the DAB-based immunohistochemistry as compared to immunofluorescence used in other cases. Since in this case, fluorescent CTB was used in one hemisphere and non-fluorescent CTB in the contralateral hemisphere, we were able to evaluate whether labeling was present only ipsilaterally or if it was also present contralaterally. In this case, labeling in the pulvinar appeared to be only ipsilateral.

In Case S2 (Figure 6), labeled axons extended from SC along the brachium, through the oral, medial and PL, as well as through central and parafascicular nuclei of the thalamus. Labeled axons were most densely concentrated in oral and PM, in the ventromedial aspect (see Figure 7D for high power magnification of an area in ventromedial PO). Retrogradely labeled cell bodies resulting from the amygdala injection were present in oral, medial and PL and the MG nucleus. Likewise, the pattern of labeling was ventromedial across the thalamus, with most cell bodies appearing in small clusters. Areas of overlap between these labeled axons and cell bodies were found within oral, medial and PL and MG. The area of most dense overlap was caudally distributed within the PM.

In summary, within the pulvinar, retrogradely labeled cell bodies were often observed in close proximity to anterogradely labeled axons. In all cases, the labeling of the cell bodies in the pulvinar after amygdala injections was sparse, suggesting it is a small projection. The areas where the retrograde label overlapped with anterograde axonal label from the SC injections were quite limited. While the oral and PM showed the largest degree of overlapping labeling, some overlap was also observed in the lateral and PI, but to a lesser degree. Confocal imaging was used to extend these observations. Figure 7 shows images within the pulvinar from cases Y1, Y2 and S2. Choleratoxin-positive cell bodies (pseudocolored red) and their processes within the pulvinar were found within a field of FE-labeled axons (pseudocolored cyan) resulting from the FE injections in SC. Representative labeling is shown in medial (Figure 7A), oral (Figures 7C,D) and inferior (Figure 7B) pulvinar. Low-power (20×) magnification shows several choleratoxin-positive cell bodies in close proximity to FE-labeled axons, while high-power (60–63×) magnification shows the processes of cell bodies and axons in close proximity to one another. As described above, labeling of cells projecting from the pulvinar to the amygdala was sparse and ventromedially concentrated, while labeling of axons from SC was ubiquitous in all images. FE-labeled axons in PM followed along the brachium, while labeling within oral and PI was more diffuse. CTB-labeled cells frequently appeared within close proximity of one another.

## Discussion

Here, we have shown a colocalization of projections from the SC with amygdala-projecting neurons in all nuclei of the macaque pulvinar, with the heaviest label occurring ventromedially in oral and PM. The overlapping distributions of retrograde and anterograde label were present in all four cases analyzed. These data provide an anatomical basis for the previously hypothesized subcortical connection between the SC and the amygdala, via the pulvinar. Since we found the same pattern of labeling in the pulvinar in two species of macaques, it is thus likely that these anatomical projections are preserved throughout this genus. While prior studies have suggested the presence of this pathway in primates through MRI-based approaches, these data provide the first direct anatomical evidence for colocalization of these projections within the primate pulvinar.

The cases we presented were tightly clustered and highly overlapping with respect to their injection sites in the amygdala and SC. Despite this there were some observable differences. For example, following the injection of the tracers in the amygdala, labeling in the PO was present in the two cases with more medial placement (Case Y2 and S2) within the lateral nucleus, and absent in the two cases with more lateral placement (Case Y1 and S1). We did not note other patterns with respect to the location of injections within the amygdala. This projection was examined in more detail by others (e.g., Stefanacci and Amaral, 2000). With respect to the injections in the SC, we noted that the two cases with more superficial injections (Y2 and S2) were associated with stronger labeling in the PO; we did not note any other patterns. As with the amygdala, the topography of projections from SC to pulvinar were examined in detail by others (Benevento and Standage, 1983; Huerta and Harting, 1983). More detailed assessment of this pathway, with injection sites aimed at the different layers of the SC and different subregions of the amygdala, should be addressed in future studies.

### Superior Colliculus Projections to the Pulvinar

In the rat and mouse, SC projects to several thalamic nuclei, including the LP nucleus (the rodent pulvinar homolog, see Harting et al., 1972) and the SG nucleus (Taylor et al., 1986; Linke et al., 1999; Zhou et al., 2017). Similarly, the SC projects to the pulvinar of the treeshrew, a proto-primate (Chomsung et al., 2008). This projection is also conserved in primates (May, 2006). In macaques, retrograde tracer injections into PM result in dense labeling of cells within the deep and intermediate layers of SC (Benevento and Standage, 1983). Moreover, lesions made in the superficial, intermediate, and deep layers of the SC result in degenerating fibers in medial and PL (Benevento and Fallon, 1975). Fibers projecting from the SC to pulvinar course laterally from the brachium and terminate throughout all of the subdivisions of the pulvinar of the squirrel monkey, owl monkey and macaque (Benevento and Fallon, 1975; Huerta and Harting, 1983; Stepniewska et al., 2000), a pattern similar to that reported here. Thus, our present findings are in line with those previously reported in the literature.

### Pulvinar Projections to the Amygdala

As with projections from the SC to the pulvinar, projections from the pulvinar to the amygdala have also been described across species. In rodents, for example, the LP nucleus projects to the lateral amygdala (Doron and LeDoux, 1999; Zhou et al., 2018). In macaques, large retrograde (horseradish peroxidase) tracer injections into the amygdala (Aggleton et al., 1980), as well as tracer injections restricted to the lateral nucleus (Norita and Kawamura, 1980; Stefanacci and Amaral, 2000), produce light labeling in the PM, primarily in a region adjacent to the brachium. In the latter study, three cases were presented in which retrograde tracers were placed in either the dorsal division or ventral intermediate division of the lateral nucleus of the amygdala. These cases displayed labeling across the rostrocaudal extend of the medial boundary of the pulvinar, a profile similar to that we observed in our cases S2 and Y2 (Figures 2, 4, 6). These projections have also been confirmed after injections of anterograde tracer into the lateral and PM in both squirrel and macaque monkeys resulted in dense terminal labeling in the dorsal division of the lateral nucleus of the amygdala (Jones and Burton, 1976; Romanski et al., 1997). More restricted injections into the medial division of the PM (i.e., along the brachium of the SC) preferentially label a narrow band at the lateral edge of the lateral nucleus of the amygdala (Burton and Jones, 1976). This pattern of labeling is consistent with what we observed in Case Y1 (Figures 2, 3).

### Convergent Labeling Within Pulvinar

In the mouse, a monosynaptic relay between the lateral SC and the lateral amygdala has been described (Wei et al., 2015). This projection is mediated by a synapse in the LP nucleus of the thalamus, the mouse homolog of the primate pulvinar (Harting et al., 1972). Interestingly, this projection is necessary for species typical responses to looming threatening stimuli (Wei et al., 2015). A similar relationship has been suggested in the rat and the treeshrew (Linke et al., 1999; Day-Brown et al., 2010). Evidence from imaging studies is consistent with these findings. In both macaque and human subjects, reports using DTI suggest that axons run from the SC to the pulvinar, and from the pulvinar to the amygdala (Rafal et al., 2015). Tamietto et al. (2012) used DTI to compare human control subjects to a patient with lesions to striate cortex. They found that probabilistic fiber bundles connecting the SC, pulvinar and amygdala exist in both lesioned and control patients, but that the projection is more robust ipsilateral to the damage in striate cortex in the lesioned subject. DTI is a probabilistic technique and cannot conclusively show either functional or anatomical coupling among the areas. Our data fill the gap between the human and primate imaging studies and the rodent data, by confirming that there is an area of overlapping projection in the pulvinar that likely connects the SC with the amygdala.

### Relevance to Behavior

Studies of blindsight have long suggested that SC is necessary for rapid visual processing, by providing an additional subcortical route for information transfer (Schneider, 1969; Weiskrantz et al., 1974). Accordingly, while lesions to striate cortex partially spare performance on visually-mediated tasks (Schmid et al., 2010), lesions or inactivation of both striate cortex and SC produce more profound deficits (Mohler and Wurtz, 1977; Solomon et al., 1981; Kato et al., 2011).

This pathway likely also serves to support rapid, non-conscious perception of threat via projections from the SC to the pulvinar, and from the pulvinar in turn, to the amygdala. Consistent with this, an increase in correlated activation among the amygdala, pulvinar, and SC is observed via fMRI when angry faces are presented in a manner that precludes conscious perception of the stimuli (Morris et al., 1999). The importance of pulvinar in this pathway is further underscored by a finding in a patient with pulvinar damage, who displays impaired fear responses to subliminally presented threatening visual stimuli (Ward et al., 2005).

Studies have also suggested that processing within the pulvinar *per se* may contribute to the rapid detection of and response to visual threats. Both humans (as measured via MRI) and macaques (as measured by single unit activity) display increased activity in the pulvinar when presented with images of snakes (Van Le et al., 2013; Le et al., 2014, 2016; Almeida et al., 2015; Soares et al., 2017). Of particular relevance to our present findings, there are two apparent clusters of snake-responsive neurons in the macaque pulvinar: one cluster in the dorsal portion of the PL, and the other in the ventromedial portion of the PM. This latter area is where we observed consistent colocalization across all four cases. In their article, Van Le et al. (2013) suggested that at least one route that could mediate rapid responses to threating stimuli such as snakes was a pathway from the SC to the pulvinar to the amygdala; our data are consistent with their interpretation.

Although it is possible that the pulvinar is relaying purely visual information to the amygdala, the intermediate and deep layers of the SC, which we have targeted in this study, receive multimodal input from different sensory systems (Stein et al., 2009). Given that other studies (Benevento and Standage, 1983) have reported preferential labeling in the intermediate and deep SC following injections into the PM, it seems likely that the labeling we observed was also due to projections from the deep and intermediate layers. This would be consistent with a multimodal relay, rather than a purely visual relay. The multimodal integration properties of the intermediate and deep layers of SC may enable its critical role for fast, reflexive defense responses (Dean et al., 1989; Brandão et al., 1994). We have recently reported that these defense responses, which are evoked by pharmacological activation of the primate SC (DesJardin et al., 2013), can be partially attenuated by concurrent inactivation of the basolateral amygdala (Forcelli et al., 2016). However, as no direct projection from SC to amygdala has been reported, it is tempting to speculate that the pulvinar is a component of the network underlying these defense responses.

In summary, here we have described a zone within the pulvinar that receives projections from the SC and contains neurons that project to the amygdala. While such a projection has been hypothesized in humans and macaques, these data provide the first direct anatomical evidence for its existence.

## Author Contributions

CE, PF, RS and LM performed experiments, edited the figures and wrote and edited the manuscript. CE analyzed the data and created the figures.

## Conflict of Interest Statement

The authors declare that the research was conducted in the absence of any commercial or financial relationships that could be construed as a potential conflict of interest.
